# Gate‐Tunable Dual‐Mode Optoelectronic Device for Self‐Powered Photodetector and Optoelectronic Synapse

**DOI:** 10.1002/advs.202416259

**Published:** 2025-03-12

**Authors:** Yi Ouyang, Chaoyi Zhang, Jun Wang, Zheng Guo, Zegao Wang, Mingdong Dong

**Affiliations:** ^1^ Interdisciplinary Nanoscience Center Aarhus University Aarhus 8000 Denmark; ^2^ Department of Biological and Chemical Engineering Aarhus University Aarhus 8000 Denmark; ^3^ School of Optoelectronic Science and Engineering University of Electronic Science and Technology of China Chengdu 610054 China; ^4^ College of Materials Science and Engineering Sichuan University Chengdu 610065 China

**Keywords:** dual‐mode, MoTe_2_/MoS_2_, optoelectronic synapse, photodetector, van der Waals heterostructure

## Abstract

In the advancing field of optoelectronics, multifunctional devices that integrate both detection and processing capabilities are increasingly desirable. Here, a gate‐tunable dual‐mode optoelectronic device based on a MoTe_2_/MoS_2_ van der Waals heterostructure, designed to operate as both a self‐powered photodetector and an optoelectronic synapse, is reported. The device leverages the photovoltaic effect in the MoTe_2_/MoS_2_ PN junction for self‐powered photodetection and utilizes trapping states at the SiO_2_/MoS_2_ interface to emulate synaptic behavior. Gate voltage modulation enables precise control of the device's band structure, facilitating seamless switching between these two operational modes. The photodetector mode demonstrates broadband detection and fast response speed, while the optoelectronic synapse mode exhibits robust long‐term memory characteristics, mimicking biological synaptic behavior. This dual functionality opens new possibilities for integrating neuromorphic computing into traditional optoelectronic systems, offering a potential pathway for developing advanced intelligent sensing and computing technologies.

## Graphical abstract

A gate‐tunable MoTe₂/MoS₂ heterostructure‐based dual‐mode optoelectronic device functions as both a self‐powered photodetector and an optoelectronic synapse. It leverages the photovoltaic effect for photodetection and trapping states for synaptic behavior. Gate modulation enables seamless switching, paving the way for integrating neuromorphic computing into optoelectronic systems for intelligent sensing and computing applications.

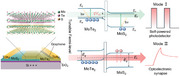

## 1 Introduction

Photodetectors are essential components in modern optoelectronic systems, with applications ranging from imaging and communication to environmental monitoring.^[^ ^1^, ^2^, ^3^ ^]^ Modern advances in this technology have led to the development of self‐powered devices, eliminating the need for external power sources.^[^ ^4^, ^5^ ^]^ These devices offer broadband detection capabilities and fast response speed, which are especially valuable in portable, low‐energy applications such as high‐speed optical communication and imaging systems.^[^ ^6^, ^7^, ^8^ ^]^ Despite these advancements, conventional photodetectors still have limitations, particularly in multifunctional applications where the integration of additional functionalities like memory and neuromorphic computing are desired.^[^ ^9^, ^10^, ^11^ ^]^

This gap has led to the development of optoelectronic synapses, inspired by biological synapses, which can both sense and process information. Unlike traditional photodetectors, which primarily focus on the detection and conversion of light signals, optoelectronic synapses can process and store information simultaneously, making them highly advantageous for applications in artificial intelligence, neuromorphic computing, and intelligent sensing.^[^ ^12^, ^13^, ^14^, ^15^, ^16^, ^17^, ^18^ ^]^ Integrating these two functionalities into a single device offers substantial benefits, including reduced device footprint, lower power consumption, and enhanced functionality.

However, merging photodetector and optoelectronic synapse mode into a single device presents challenges, particularly in achieving tunable and reversible switching between these modes. Several strategies have been explored to achieve dual‐mode functionality in optoelectronic devices, including tuning the source–drain voltage (*V*_ds_),^[^ ^19^, ^20^, ^21^ ^]^ gate voltage (*V*_g_),^[^ ^22^, ^23^, ^24^ ^]^ and device configuration.^[^ ^25^ ^]^ Previous designs, however, have faced significant limitations. For instance, the photodetector mode may suffer from narrow detection bands, slow detection speeds, and limitations in self‐powered operation. In addition, the optoelectronic synapse modes can struggle with low sensitivity and inadequate signal‐to‐noise ratios. To successfully integrate these technologies, innovative solutions are needed to overcome these challenges and optimize both functionality and performance.

**Figure 1:**
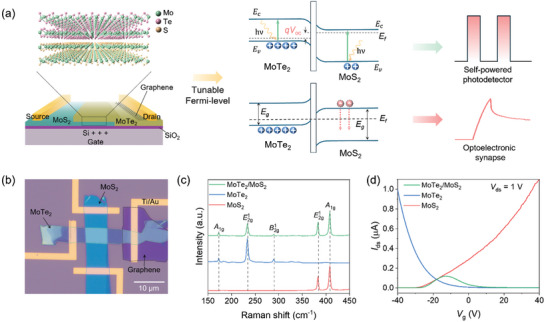
Characterizations of MoTe_2_/MoS_2_ vdWH. a) A schematic representation of the MoTe_2_/MoS_2_ vdWH, highlighting its dual application as a self‐powered photodetector and an optoelectronic synapse. b) Optical microscopy image of the fabricated MoTe_2_/MoS_2_ vdWH. c) Raman spectra of MoTe_2_, MoS_2_, and MoTe_2_/MoS_2_ vdWH. d) Transfer curves of MoTe_2_, MoS_2_, and MoTe_2_/MoS_2_ vdWH.

In this work, we propose a novel dual‐mode optoelectronic device based on a MoTe_2_/MoS_2_ Van der Waals heterostructure (vdWH). The dual‐mode functionality is achieved by tuning the *V*_g_, allowing it to switch between photodetector and optoelectronic synapse modes (**Figure** **1**a). The p‐type MoTe_2_ and n‐type MoS_2_ form a PN junction, enabling the device to function as a self‐powered photodetector through the photovoltaic effect. On the other hand, when the *V*_g_ depletes the MoS_2_ layer, the device operates as an optoelectronic synapse, with the mechanism attributed to the trapping states at the SiO_2_/MoS_2_ interface. This dual‐mode operation not only enhances the versatility of the device but also opens new avenues for integrating neuromorphic computing with conventional optoelectronics.

## 2 Results and Discussion

Figure 1b presents an optical image of the MoTe_2_/MoS_2_ vdWH fabricated using a polycarbonate dry‐transfer method.^[^ ^26^ ^]^ To facilitate efficient hole injection, a multi‐layer graphene was transferred onto the MoTe_2_ flake, serving as the contact material.^[^ ^27^, ^28^, ^29^ ^]^ The metal electrodes were then patterned using electron‐beam (e‐beam) lithography, followed by e‐beam evaporation of Ti (3 nm) and Au (35 nm) to form metal contacts. Figure 1d displays the Raman spectra of the 2H‐phase MoS_2_, MoTe_2_, and the as‐fabricated heterostructure. In the MoS_2_ spectrum (red line), peaks at 384 and 409 cm^−1^ correspond to the in‐plane vibration mode ($E_{2g}^{1}$) and out‐of‐plane vibration mode, respectively. The peaks located at 173, 234, 290 cm^−1^ represent the out‐of‐plane mode (*A_1_* *_g_*), in‐plane mode ($E_{2g}^{1}$), and in‐active phonon mode ($B_{2g}^{1}$) of MoTe_2_ (blue line), respectively. These peaks are also observed in the heterostructure region (green line), confirming the formation of a high‐quality vdWH.^[^ ^30^, ^31^ ^]^ The Raman analysis verifies that the graphene used for contact is of a multilayer nature (Figure S1, Supporting Information). The transfer curves of MoS_2_, MoTe_2_ and MoS_2_/MoTe_2_ vdWH are shown in Figure 1d, with the *V*_ds_ set to 1 V. As expected, MoS_2_ and MoTe_2_ exhibit distinct n‐type and p‐type^[^ ^32^ ^]^ behaviors, respectively. The transfer curve of MoTe_2_/MoS_2_ vdWH shows an overall anti‐ambipolar shape, which can be qualitatively understood by considering the series resistance of MoS_2_ and MoTe_2_.^[^ ^33^, ^34^ ^]^ When the *V*_g_ is below −25 V or above 5 V, either MoS_2_ or MoTe_2_ approaches the off‐state, resulting in a low drain–source current (*I*_ds_). The use of multilayer graphene as the contact material for MoTe_2_ significantly reduces the Schottky barrier height, facilitating more effective hole injection. This effect becomes apparent when comparing the transfer characteristics of devices with and without graphene contacts (Figure S2, Supporting Information). The high Schottky barrier in the absence of graphene results in lower peak currents and a less pronounced anti‐ambipolar behavior.

**Figure 2:**
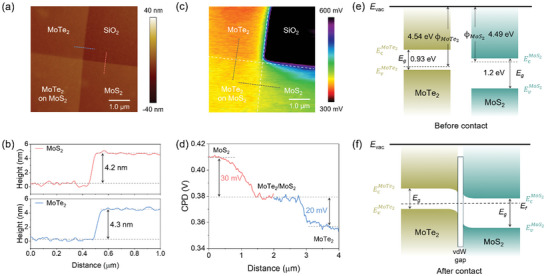
AFM, KPFM, and energy band alignment of the MoTe_2_/MoS_2_ vdWH. a) AFM image of the as‐fabricated MoTe_2_/MoS_2_ vdWH. b) The height profiles of MoS_2_ and MoTe_2_, respectively. c) The KPFM image of the MoTe_2_/MoS_2_ vdWH in the same area. d) The CPD between MoS_2_ and MoTe_2_. e,f) Energy band diagrams of the MoTe_2_/MoS_2_ vdWH, both before (e) and after contact (f).

**Figure** **2**a presents the atomic force microscope (AFM) image of MoTe_2_/MoS_2_ vdWH. The thickness of MoS_2_ and MoTe_2_ are estimated to be 4.2 nm (about 6 layers) and 4.3 nm (about 6 layers), respectively, based on the red and blue scan line profiles shown in Figure 2b. Kelvin probe force microscopy (KPFM) was conducted on the same area to measure the work function of MoS_2_ and MoTe_2_ (Figure 2c), clearly illustrating the surface potential distribution in the heterostructure region. Through locally extracting line profile, the contact potential difference (CPD) between MoS_2_ and MoTe_2_ is illustrated on Figure 2d. The surface potential difference between MoS_2_ and MoTe_2_ is estimated to 50 meV. The work function of materials is highly related to their substrate materials, leading to a 20 meV surface potential difference between MoTe_2_ on SiO_2_ substrates and on MoS_2_ flakes.^[^ ^35^, ^36^, ^37^, ^38^ ^]^ For a quantitative analysis, the work function of the 2D materials can be expressed as:

(1) $$\Phi_{2D}=\Phi_{probe}-\;eV_{CPD}$$

where Φ_2*D*_, Φ_*probe*_, and *V_CPD_* are the work function of 2D material, probe (estimated at 4.9 eV^[^ ^39^ ^]^) and voltage bias applied to the probe to compensate the CPD, respectively. Using this relation, the work functions of MoS_2_ and MoTe_2_ are calculated to be 4.49 and 4.54 eV, respectively. These values also correspond well with the previous report that use multi‐layer MoS_2_ and MoTe_2_ for heterostructure.^[^ ^35^ ^]^ Based on the KPFM data, energy band diagrams of the MoTe_2_ and MoS_2_ before and after contact are constructed, as depicted in Figure 2e. The bandgaps of MoTe_2_ and MoS_2_ are taken as 0.93 and 1.2 eV, respectively, forming a type II (staggered) heterostructure.^[^ ^40^, ^41^ ^]^ Upon contact, thermal equilibration leads to band bending at the interface, creating a built‐in potential barrier within a thin van der Waals junction gap region (Figure 2f). Depending on the applied *V*_g_, the MoTe_2_/MoS_2_ vdWH divides into two photoresponse mode, that is, self‐powered photodetector and artificial optoelectronic synapse, as discussed below.

**Figure 3:**
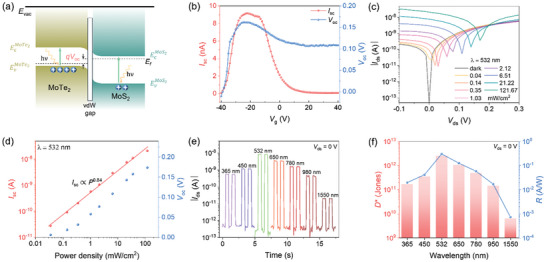
Self‐powered photodetector performance of the MoTe_2_/MoS_2_ vdWH. a) Band alignment of the MoTe_2_/MoS_2_ vdWH working as a photovoltaic device under illumination. b) *V*_oc_ and *I*_sc_ of the device at varying *V*_g_, with *P* = 60 mW cm^−2^ and λ = 532 nm. c) Logarithmic |*I*_ds_|–*V*_ds_ curves of the device under dark conditions and at various *P*, λ = 532 nm. d) *V*_oc_ and *I*_sc_ as a function of *P* under 532 nm light. e) Time‐dependent photoresponse behavior of the device under different wavelengths light at *P* = 60 mW cm^−2^, *V*_ds_ = 0 V. f) Wavelength‐dependent D* and *R* of the device.

The capability of the device as a self‐powered photodetector stems from its photovoltaic effect, as depicted in **Figure** **3**a. When illuminated, the photoexcited holes and electrons move to the p‐side and n‐side, respectively, generating the open‐circuit voltages (*V*_oc_) and short‐circuit currents (*I*_sc_).^[^ ^42^, ^43^, ^44^ ^]^ Figure 3b summarizes the *V*_oc_ and *I*_sc_ of the device at various *V*_g_. The scanning photocurrent mapping under different *V*_g_ (−30, −20, and 0 V) confirms that the changes in *V*_oc_ and *I*_sc_ are predominantly caused by doping modulation of the MoTe_2_ and MoS_2_ layers in the heterojunction region, as shown in Figure S3 (Supporting Information). The *V*_g_ alters the built‐in electric field in the heterojunction, which drives the photovoltaic effect. In contrast, no obvious photocurrent was observed at the metal–semiconductor contact regions. The optimal photovoltaic performance of the device under 532 nm illumination is observed at *V*_g_ ≈ −20 V, which can be attributed to the modulation of energy band alignment at the MoTe_2_/MoS_2_ vdWH. At this *V*_g_, the built‐in electric field at the junction is maximized, enabling efficient separation and transport of photogenerated carriers. Deviations from this optimal gate voltage lead to either excessive carrier accumulation or depletion, reducing the photovoltaic efficiency. The optimal *I*_sc_ and *V*_oc_ are observed at *V*_g_ = −20 V, so *V*_g_ is fixed at −20 V for further photovoltaic performance evaluations. Figure 3c presents the *I*_ds_–*V*_ds_ curves on logarithmic scales of the device under dark and 532 nm illumination with varying power density (*P*). The photocurrent (*I*_ph_) increases monotonically with *P*. The corresponding time‐dependent photoresponse is detailed in Figure S4 (Supporting information). Owing to the photovoltaic effect, the device achieves an illumination on/off ratio approaching 10^5^, with a peak *I*_ph_ of 20 nA. The relationship between the *P* and *I*_ph_ is described by the equation:

(2) $$I_{\mathrm{ph}}=AP^{\alpha }\left(0<\alpha <1\right)$$

where *A* and α are constant and a fitting index, respectively. The value of α is calculated to be 0.84, as shown in Figure 3d, and a linear relationship between *V*_oc_ and *P* confirms the excellent photovoltaic effect of the device.^[^ ^45^ ^]^ Figure 3e shows the |*I*_ds_|–*V*_ds_ characteristics under various wavelengths at *P* = 60 mW cm^−2^, from ultraviolet to infrared, highlighting the device's stable and broad photodetection range. The broadband photodetection performance of the device is attributed to its inherent optical absorption characteristics, which encompass a wide spectral range extending from the visible to the near‐infrared region.^[^ ^43^ ^]^ This characteristic has been corroborated by prior studies, as detailed in Table S1 (Supporting Information). The response time of the photodetector is critical for performance assessment. The normalized time‐resolved photoresponse of the device is shown in Figure S5 (Supporting Information). The rise time (τ_r_) and fall time (τ_f_) are defined as the time interval of the photocurrent rise from 10% to 90% and decay from 90% to 10%, respectively. The representative τ_r_ and τ_f_ of MoTe_2_/MoS_2_ vdWH are 188 µs and 45 µs, respectively, reflecting a rapid photoresponse due to effective carrier generation and separation.^[^ ^44^ ^]^ The photodetection performance is quantitatively evaluated through responsivity (*R*) and specific detectivity (D*), calculated as follows:

(3) $$R=\left(I_{\mathrm{ph}}-I_{\mathrm{dark}}\right)/\left(P\cdot S\right)$$

(4) $$D^{*}=R\cdot S^{1/2}/{\left(2e\cdot I_{\mathrm{dark}}\right)}^{1/2}$$

where *I*_dark_, *S*, and *e* represent dark current, effective area, and the charge of an electron, respectively.^[^ ^46^ ^]^ The effective area of the device is defined as the overlapped region of the MoTe_2_/MoS_2_ vdWH that responds to photons and generates the photovoltaic effect. Figure 3f presents the calculated *R* and D* at different wavelengths under 60 mW cm^−2^, with maximum values of 0.3 A W^−1^ for *R* and 2.56 × 10^12^ Jones for D* observed at 532 nm illumination. Table S1 (Supporting Information) compares the performance of our MoTe_2_/MoS_2_ vdWH based self‐powered photodetector with recent reports, including metrics like thickness, *V*_oc_, photoresponse range, *R*, and response speed. Our device demonstrates broadband detection, fast response, and high responsivity, highlighting its competitive performance and the effectiveness of our heterostructure design.

**Figure 4:**
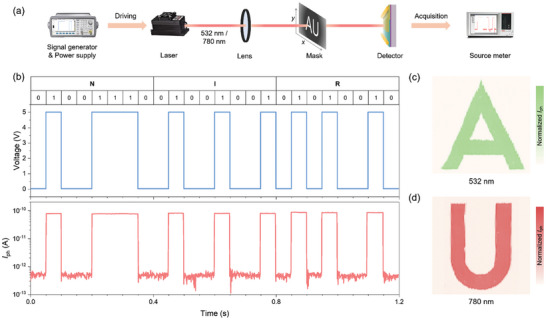
Applications of MoTe_2_/MoS_2_ vdWH in single‐device optical communication and imaging system. a) Schematic diagram of setups for the functional applications. b) The input and output signals transmitted using the ASCII code “NIR” through the optical communication system. d,e) The output images obtained from a mask with letters “A” and “U”, captured by a single‐pixel imager under 532 and 780 nm illumination, respectively. All measurements were conducted at *V*_g_ = −20 V, ensuring optimal device performance.

The MoTe_2_/MoS_2_ vdWH exhibits exceptional broadband photodetection and fast response speed, showcasing its potential for diverse optoelectronic applications. **Figure** **4**a depicts a schematic of a single‐device optical communication and imaging system. A signal generator drives and controls the input signal from a laser source for various applications. A glass shadow mask, marked with the letters “A” and “U” is mounted on a two‐axis planar moving stage to function as a shutter to direct light onto the detector. Then the photocurrent from the device is recorded and transformed into ASCII code and images. Figure 4b demonstrates the generation of binary data stream containing the data information “NIR”. These optical signals are coded through the signal generator, received by the device and converted into the corresponding letter. The device's rapid photoresponse and high on/off ratio ensure the precision and reliability of optical communication. Images of the letters “A” and “U” were constructed using wavelengths of 532 and 780 nm, as displayed in Figure 4c,d, respectively. Both images exhibit well‐defined edges and high contrast, indicating the system's precision and resolution. The clear demarcation of the letter boundaries and strong contrast of the images highlight the system's precision and high‐resolution capabilities.

**Figure 5:**
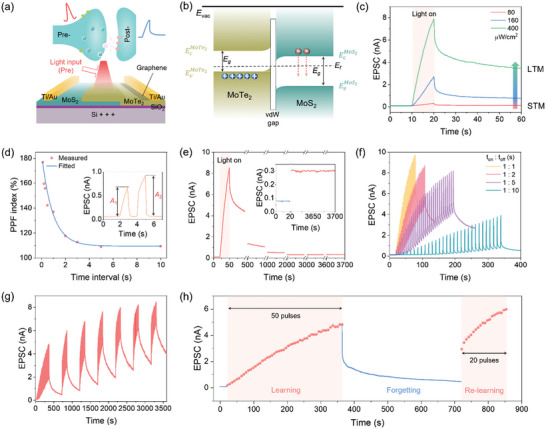
The performance of the MoTe_2_/MoS_2_ vdWH as an optoelectronic synapse. a) Schematic diagram of a biological synapse and experimental setup. b) Band alignment of MoTe_2_/MoS_2_ vdWH working as an optoelectronic synapse under illumination. c) The transition from STM to LTM behavior by varying light pulse intensity. d) PPF index as a function of time interval. The inset shows the light‐induced EPSC and the definition of *A*_1_ and *A*_2_ with a time interval of 1 s. e) The retention time of EPSC. Inset: the comparison between the initial current and EPSC after 1 h. f) The EPSC induced by a series light pulse with different light pulse on/off ratio, *P* = 160 µW cm^−2^. g) The EPSC response under seven light pulse sequences. h) “Learning‐experience” behavior under pulse stimulation. All the characterizations were conducted at *V*_g_ = −40 V and *V*_ds_ = 1 V.

The Fermi level of the vdWH is finely tuned by applying different *V*_g_, which enables the transition between photodetector and optoelectronic synapse modes. The *V*_g_ modulates the charge carrier dynamics in both MoS_2_ and MoTe_2_ layers, with the dominant effect observed in MoS_2_. At negative V_g_, MoS_2_ undergoes significant depletion, facilitating carrier interaction with defect states at the MoS_2_/SiO_2_ interface. MoTe_2_ contributes to the heterojunction by providing a staggered band alignment, stabilizing the built‐in electric field, and enabling efficient hole injection, which collectively enhance the optoelectronic synapse performance. The performance of MoTe_2_/MoS_2_ vdWH as an optoelectronic synapse was systematically evaluated. As schematically illustrated in **Figure** **5**a, the device is illuminated by light pulses to simulate the external stimuli. The device acts as an artificial synapse, converting light stimulation into excitatory postsynaptic current (EPSC) to replicate the signal transmission between pre‐synaptic and post‐synaptic neurons in biological synapses. When MoS_2_ is fully depleted (*V*_g_ = −40 V), light illumination generated electron‐hole pairs increase the carrier concentration in MoS_2_ significantly, thereby enhancing the overall conductivity of the device under bias. Continuous illumination leads to the accumulation of photogenerated electrons in the trapping states between MoS_2_ and the SiO_2_ interface, resulting in a consistent increase in *I*_ds_. After illumination, the electrons stored in the potential well gradually return to the valence band over time, causing *I*_ds_ to revert to the original level (Figure 5b).^[^ ^15^, ^22^, ^47^ ^]^ Figure 5c shows the optoelectronic synapse function of the device under 532 nm light with various *P* ranging from 80 to 400 µW cm^−2^. As the *P* increases, the EPSC rises significantly, indicating the transition from short‐term memory (STM) to long‐term memory (LTM).^[^ ^48^, ^49^, ^50^ ^]^ The decay rate of the EPSC, assessed by normalizing the *I*_ds_ decay curves (Figure S6, Supporting Information), shows a clear inverse correlation with *P*. The impact of varying wavelengths on the optoelectronic synapse performance was evaluated by testing the device under illumination at 405, 532, and 780 nm (Figure S7, Supporting Information). The results show similar synaptic behavior across all tested wavelengths, indicating that the synaptic performance is primarily governed by the trapping and de‐trapping dynamics at the MoS_2_/SiO_2_ interface and is not significantly influenced by the photon energy within this range. The role of defect states at the MoS_2_/SiO_2_ interface in inducing optoelectronic synapse properties was confirmed through comparative experiments (Figure S8, Supporting Information). When the vdWH was isolated from the SiO_2_ substrate using an hBN layer and operated with a graphene/hBN top‐gate structure, the device exhibited only photovoltaic performance, with no evidence of synaptic behavior.

Paired pulse facilitation (PPF) is a fundamental aspect of short‐term plasticity (STP), playing a crucial role in visual information processing in biological systems. Figure 5d depicts the PPF index as a function of light pulse interval, defined by the equation:

(5) $$\mathrm{PPF}=A_{2}/A_{1}\times 100\%$$

where 𝐴_2_ represents the EPSC *generated* by the second pulse and 𝐴_1_ by the first. As a pair of light pulses illuminated to the device, the EPSC generated by *A*_2_ is noticeable higher than *A*_1_, as shown in the inset of Figure 5d. This current accumulation phenomenon is attributed to the increased photo‐induced carrier concentration embedded into the trapping states in MoS_2_. These electrons relax to the initial state over time, causing a reduction in EPSC accumulation and a corresponding decrease in PPF with longer intervals between pulses. Furthermore, the PPF decay trend is well described by a double exponential decay function (blue line in Figure 5d), suggesting the device's resemblance to biological synapses.

Long‐term plasticity (LTP), crucial for memory formation, involves transitioning from STM to LTM through repeated practice. Increasing pulse width significantly enhances EPSC, as shown in Figure S9 (Supporting Information), with longer pulse widths requiring more time for EPSC to return to its initial state, indicating the shift from STP to LTP. For pulse widths of 30 s, LTP retention exceeds 1 h, as depicted in Figure 5e. The ratio of EPSC after 1 h to the initial current exceeds 3 (inset image in Figure 5e), indicating the robustness and stability of the induced LTP. This observation is consistent with the temporal scale of synaptic potentiation observed in biological systems. Figure 5f presents EPSC recorded at varying pulse frequencies while maintaining a constant pulse width of 1s. The interval between consecutive pulses was systematically varied, ranging from 1s to 10s. As the number of pulses increases, there is a notable and proportional rise in the EPSC values, demonstrating the device's capability to emulate biological synaptic behavior, specifically spike‐rate‐dependent plasticity (SRDP). SRDP is a neural mechanism where synaptic strength is modulated by the rate of presynaptic neuron firing. The experimental data effectively replicates this mechanism, with EPSC amplitude increasing with pulse frequency, simulating how biological synapses strengthen with repeated stimulation. Similar EPSC responses were observed with varying light intensities at a fixed pulse frequency, as shown in Figure S10 (Supporting Information). The gate‐tunable dual‐mode functionality of the MoTe_2_/MoS_2_ vdWH is confirmed by measurements on additional devices. These devices exhibit a pronounced photovoltaic effect and characteristic EPSC responses under light stimulation, as shown in Figure S11 (Supporting Information).

Figure 5g illustrates the EPSC response to a sequence of light pulses designed to simulate the human “learning‐experience” behavior. This process typically involves three phases: learning, forgetting, and re‐learning. During the “learning” phase, the device is stimulated by 50 consecutive light pulses, leading to a gradual increase in EPSC. Following the cessation of light pulses, EPSC gradually declines, representing the “forgetting” phase. In the “re‐learning” phase, the device is subjected to a shorter series of 20 light pulses (Figure 5h). Notably, the EPSC recovers more rapidly, restoring and even surpassing the previous learning level. This accelerated recovery highlights the device's ability to relearn more efficiently, similar to the human brain's improved capacity to retain information after initial learning, thanks to the residual changes from the first learning phase.

**Figure 6:**
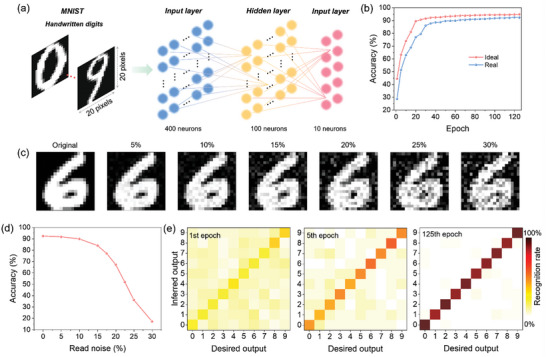
Application of MoTe_2_/MoS_2_ vdWH in image recognition. a) Schematic diagram of MLP simulator for image recognition of MNIST database. b) MNIST image classification accuracy based on ideal device and MoTe_2_/MoS_2_ vdWH (real), respectively. c) Examples of MNIST images presented with varying levels of noise. d) Classification accuracy as a function of noise level. e) The confusion matrix between desired value and predicted value after 1st, 5^th^, and 125th training epoch.

To evaluate the learning performance of the device in optoelectronic neural network, a multi‐layer perceptron (MLP) simulator was constructed to verify its accuracy in classifying Modified National Institute of Standards and Technology (MNIST) handwritten digit images (**Figure** **6**a).^[^ ^51^, ^52^ ^]^ The MNIST dataset comprises images of digits from 0 to 9. For this analysis, these images were resized from their original 28 × 28 pixels to 20 × 20 pixels by cropping the edges. The neural network is structured with 400 input neurons, corresponding to the number of pixels, 100 hidden neurons, and 10 output neurons, each representing a digit class. During training, the simulator processes 8000 randomly selected images from the 60000‐image training set. The network's weights were adjusted using feedback from the adaptive moment estimation (Adam) optimizer. In the test process, the network's performance was assessed by classifying 10000 images from the test set. The device demonstrated a peak classification accuracy of 92.3%, as shown in Figure 6b. This result is notably close to the ideal device's maximum classification accuracy, which stands at 94.8%. The device's recognition accuracy was also tested under varying levels of read noise. Figure 6c shows a series of sample images with varying noise levels, ranging from original to 30%. The accuracy under different read noise conditions is shown in Figure 6d and Figure S12 (Supporting Information). It is observed that the device maintains high accuracy at low noise levels, but the accuracy sharply decreases with increased noise levels. Figure 6e displays the confusion matrices that illustrate the recognition accuracy during the 1st, 5th, and 125th epochs of the learning process.^[^ ^53^, ^54^ ^]^ As the training progresses, the accuracy improves significantly. The accuracy improves significantly as training progresses, with the model's inferred output closely aligning with the desired outputs by the 125th epoch. This alignment suggests that the model effectively learns to classify the data, reducing errors and improving overall performance as the training continues.

## 3 Conclusion

In this work, we introduced an innovative gate‐tunable dual‐mode optoelectronic device based on a MoTe_2_/MoS_2_ heterostructure that functions as both a self‐powered photodetector and an optoelectronic synapse. By modulating the *V*_g_, we achieved tunable and reversible switching between these two modes. The self‐powered photodetector mode, enabled by the photovoltaic effect in the MoTe_2_/MoS_2_ PN junction, exhibited excellent broadband photodetection and fast response speed, making it highly suitable for applications in optical communication and imaging. Conversely, the optoelectronic synapse mode, facilitated by the trapping states at the SiO_2_/MoS_2_ interface, effectively emulated synaptic behaviors such as short‐term plasticity, long‐term plasticity, and spike‐rate‐dependent plasticity. The device's ability to simulate biological learning and forgetting processes was further validated through an optoelectronic neural network, achieving a high classification accuracy in handwritten digit recognition tasks. These results highlight the significant potential of our device for advancing neuromorphic computing systems and multifunctional optoelectronic applications. The integration of self‐powered photodetection with neuromorphic computing in a single device marks a significant advancement in optoelectronics, paving the way for the development of intelligent and energy‐efficient sensing systems.

## 4 Experimental Section

### Device Fabrication

The MoS_2_ flakes, MoTe_2_ flakes, and graphene flakes were exfoliated using Scotch tape and transferred onto 285 nm SiO_2_/Si substrates. The MoTe_2_/MoS_2_ heterostructures with multi‐layer graphene as contact electrode for MoTe_2_ were stacked and transferred onto SiO_2_/Si substrates by polycarbonate dry‐transfer method. The contact metal electrodes with 3 nm Ti/35 nm Au were patterned by e‐beam lithography and deposited by e‐beam deposition.

### Characterizations and Measurements of Photodetectors

The Raman spectroscopy was measured on Renishaw platform with a laser of 514 nm. The AFM and KPFM images were characterized by the scanning probe microscope (Asylum Research Cypher). The tip was kept 50 nm above the sample during the KPFM characterization. The optoelectronic characteristics of the device were tested by Keithley 4200s semiconductor analyzer and commercial semiconductor laser sources. The scanning photocurrent mapping was conducted using a Raman–atomic force microscope (Alpha300RA) under 532 nm illumination.

## Conflict of Interest

The authors declare no conflict of interest.

## Supporting information



Supporting Information

## Data Availability

The data that support the findings of this study are available from the corresponding author upon reasonable request.
