# Supplementary material for: Gate‐Tunable Dual‐Mode Optoelectronic Device for Self‐Powered Photodetector and Optoelectronic Synapse
Source: Adv Sci (Weinh). 2025 Mar 12;12(17):2416259. doi: 10.1002/advs.202416259 (PMC12061284; doi:10.1002/advs.202416259)
Supplement: Supplementary file 1 — Supporting Information [file ADVS-12-2416259-s001.docx]

Supporting Information:

Gate-tunable Dual-mode Optoelectronic Device for Self-powered Photodetector and Optoelectronic Synapse

*Yi Ouyang^1,2^, Chaoyi Zhang^1,3^, Jun Wang^3^, Zheng Guo^2,^*, Zegao Wang^4,^*, Mingdong Dong^1,^**

^1^ Interdisciplinary Nanoscience Center, Aarhus University, Aarhus 8000, Denmark.

^2^ Department of Biological and Chemical Engineering, Aarhus University, Aarhus 8000, Denmark.

^3^ School of Optoelectronic Science and Engineering, University of Electronic Science and Technology of China, Chengdu 610054, China

^4^ College of Materials Science and Engineering, Sichuan University, Chengdu 610065, China.

*Corresponding to: dong@inano.au.dk, zegao@scu.edu.cn, guo@bce.au.dk


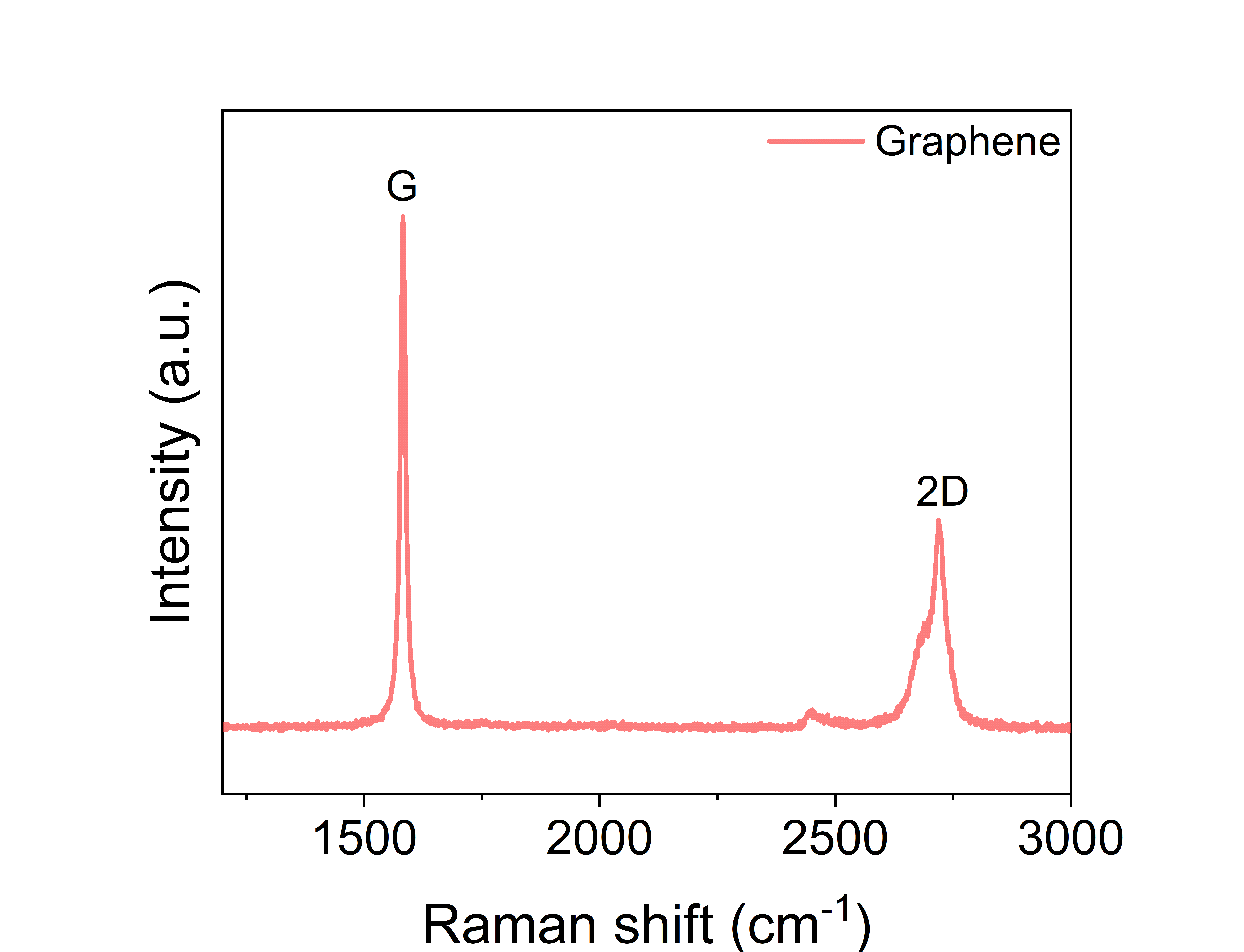


Figure S1. Raman spectra of multi-layer graphene.


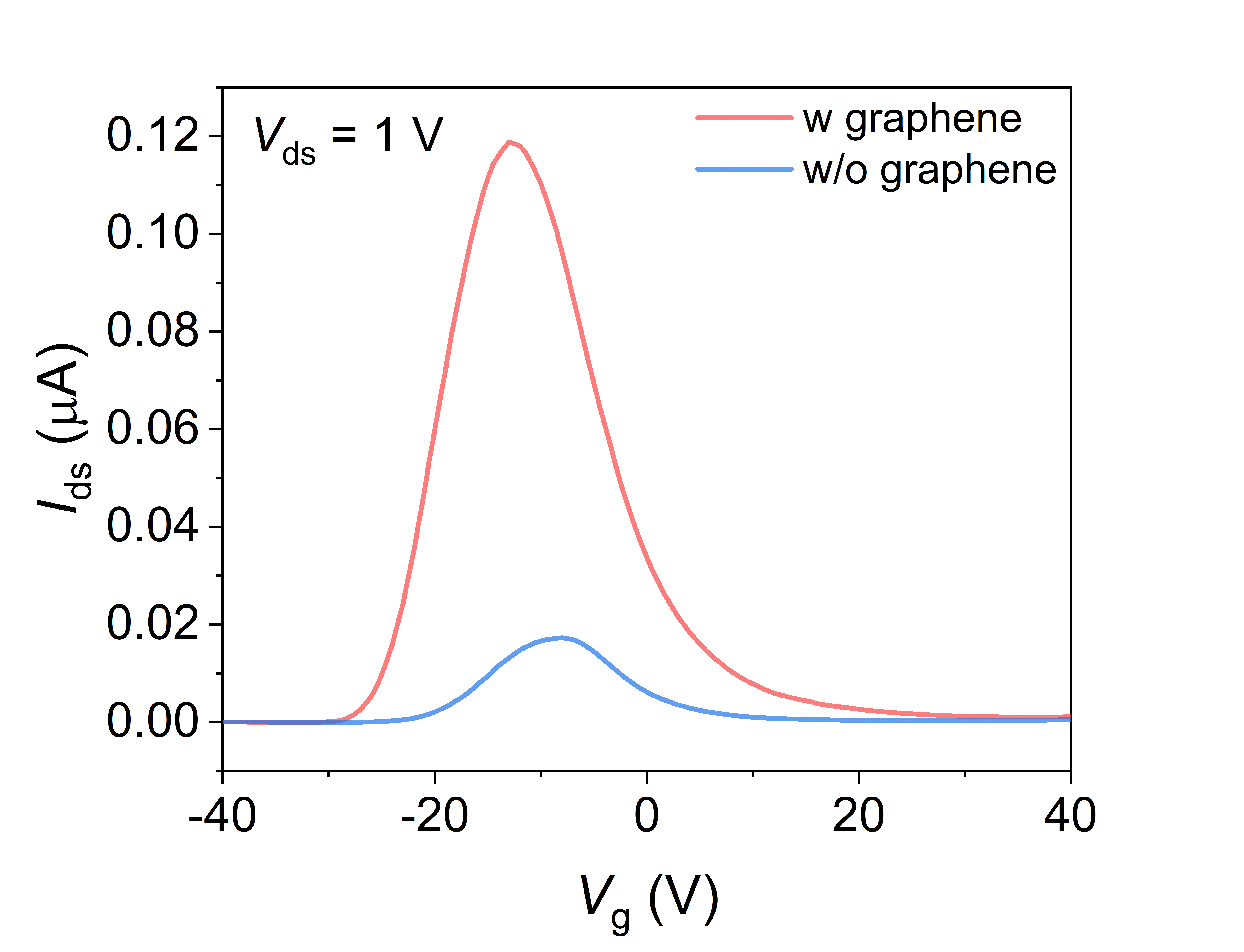


Figure S2. Transfer curve of MoTe_2_/MoS_2_ Van der Waals heterostructure (vdWH) with (w) and without (w/o) graphene.


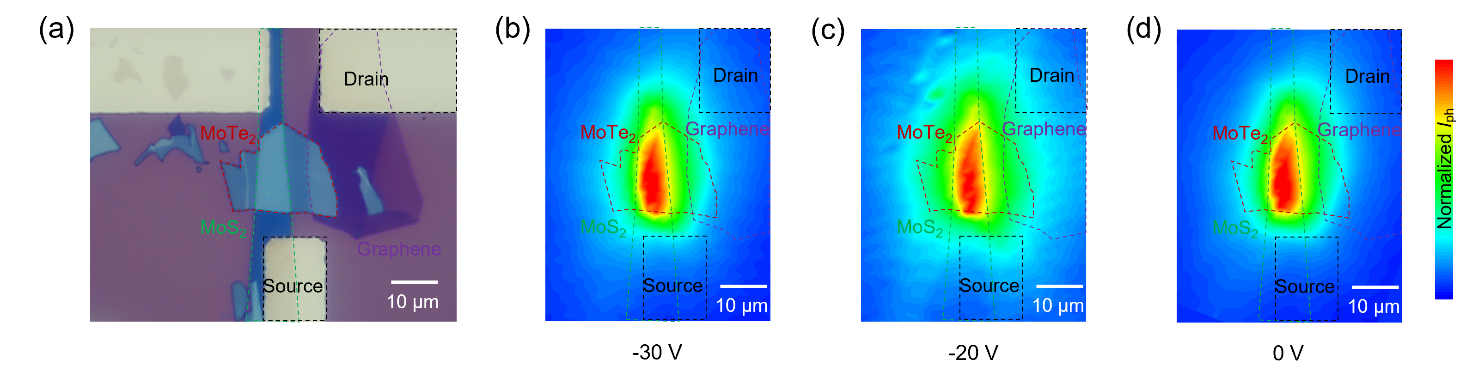


Figure S3. The scanning photocurrent mapping of the device under 532 nm illumination with different *V*_g_ (-30 V, -20 V, and 0 V) at *V*_ds_ = 0 V.


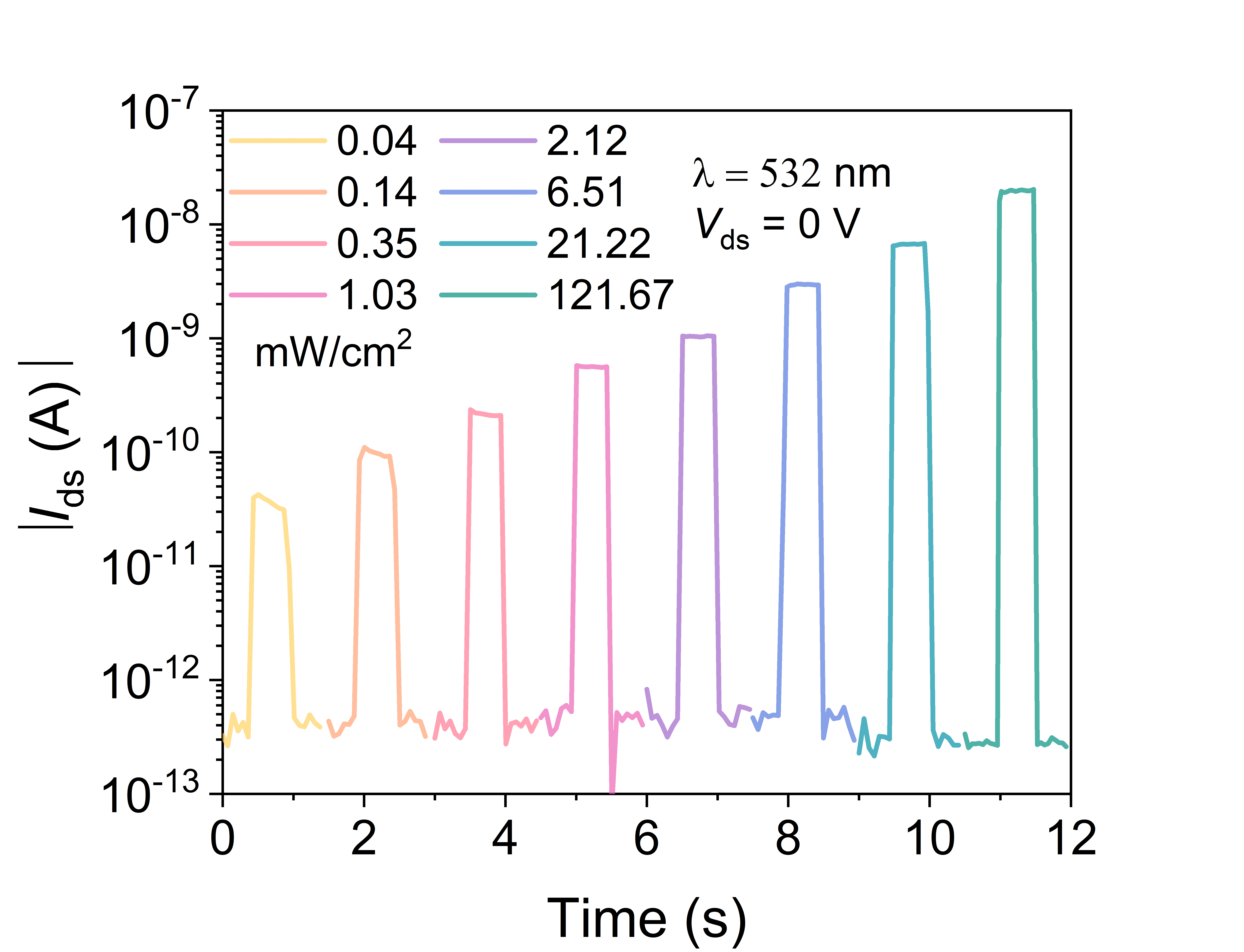


Figure S4. Time-dependent photoresponse of the MoTe_2_/MoS_2_ vdWH under 532 nm illumination with different power densities.


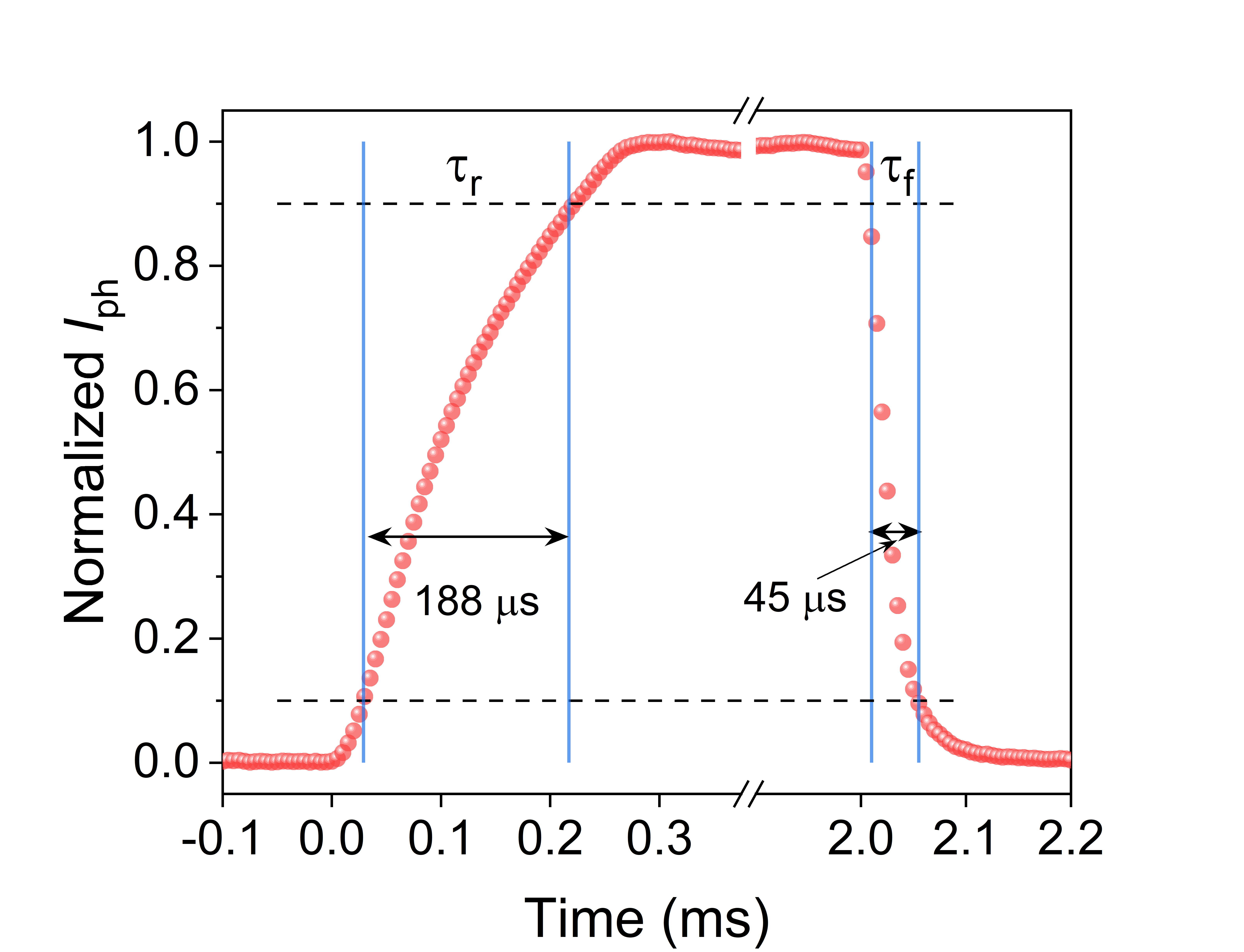


Figure S5. The normalized time-resolved photoresponse of the device.


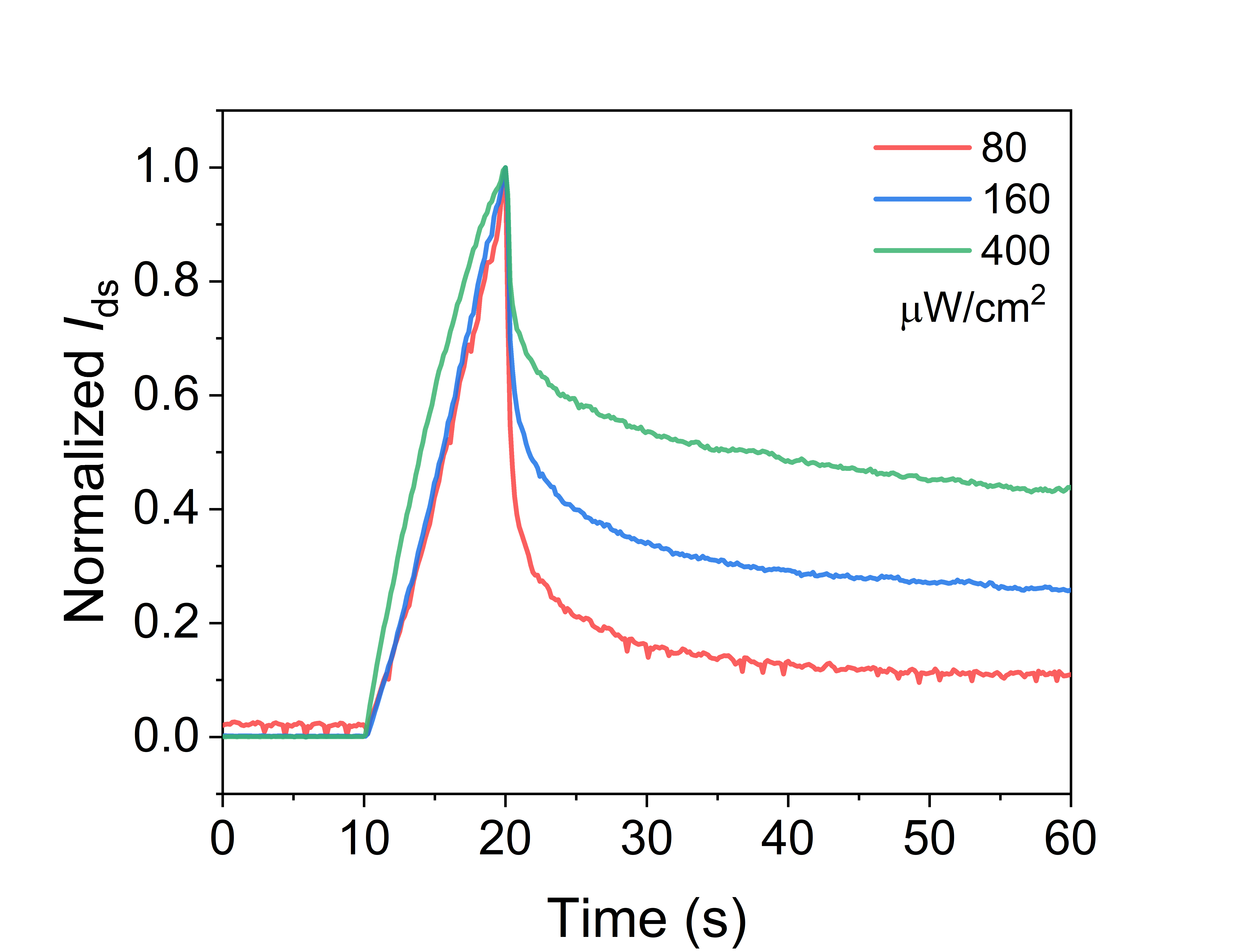


Figure S6. The normalized *I*_ds_ decay speed under different 532 nm light power density.


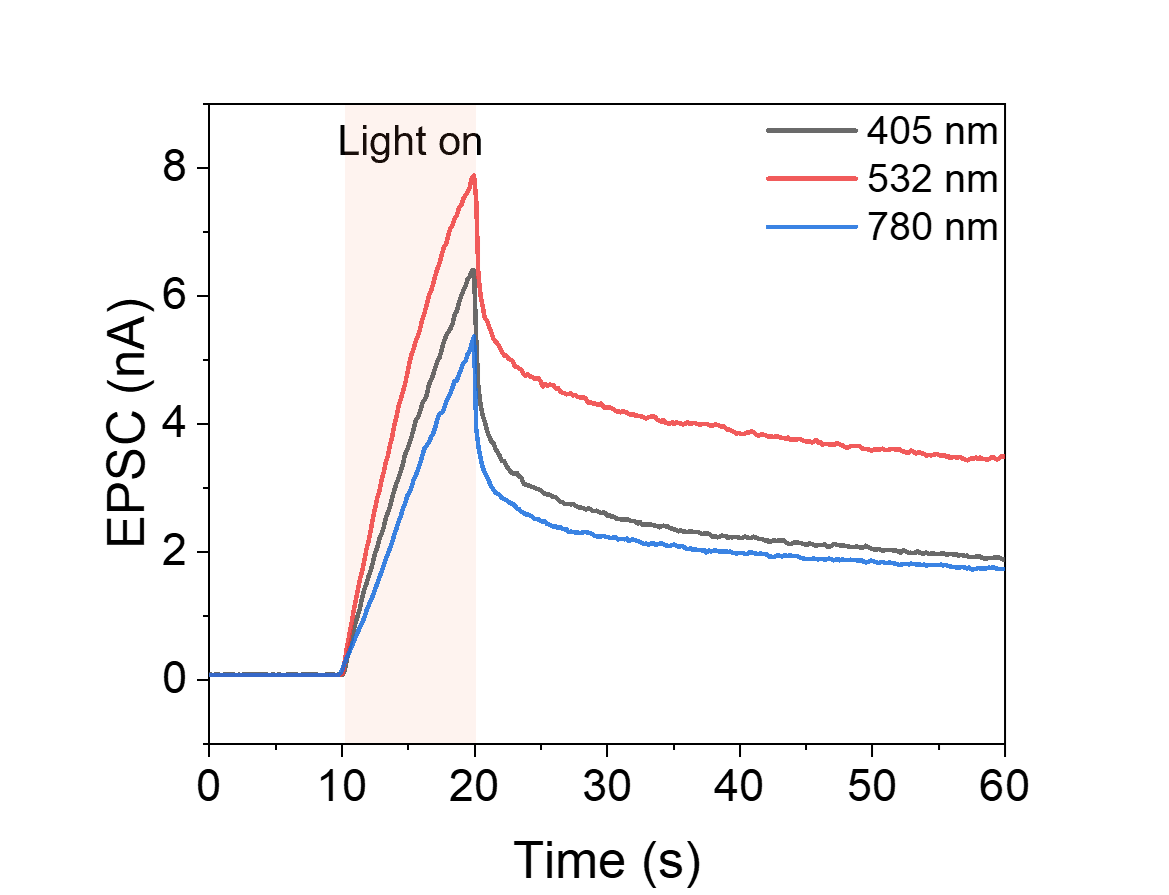


Figure S7. The optoelectronic synapse performance of the device under illumination at 405 nm, 532 nm, and 780 nm with *P* = 160 µW/cm^2^.


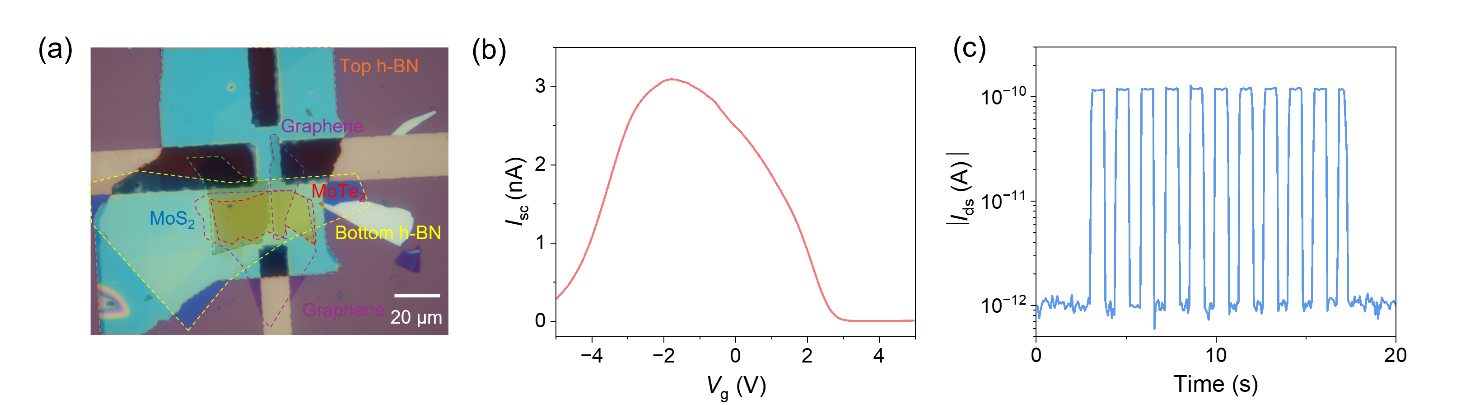
Figure S8. (a) Optical microscopy image of a top-gated MoTe_2_/MoS_2_ vdWH isolated from the SiO_2_ substrate through hBN. (b) *I*_sc_ of the device at varying *V*_g_, with λ = 532 nm. (c) Time-dependent photoresponse behavior of the device under 532 nm light with *V*_ds_ = 1 V and *V*_g_ = -4 V.


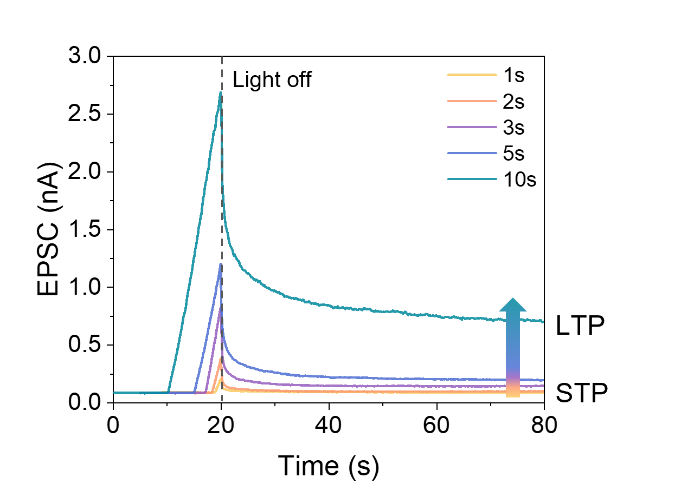


Figure S9. EPSC response under light power of 160 µW/cm^2^ with light pulse width from 1 s to 10 s.


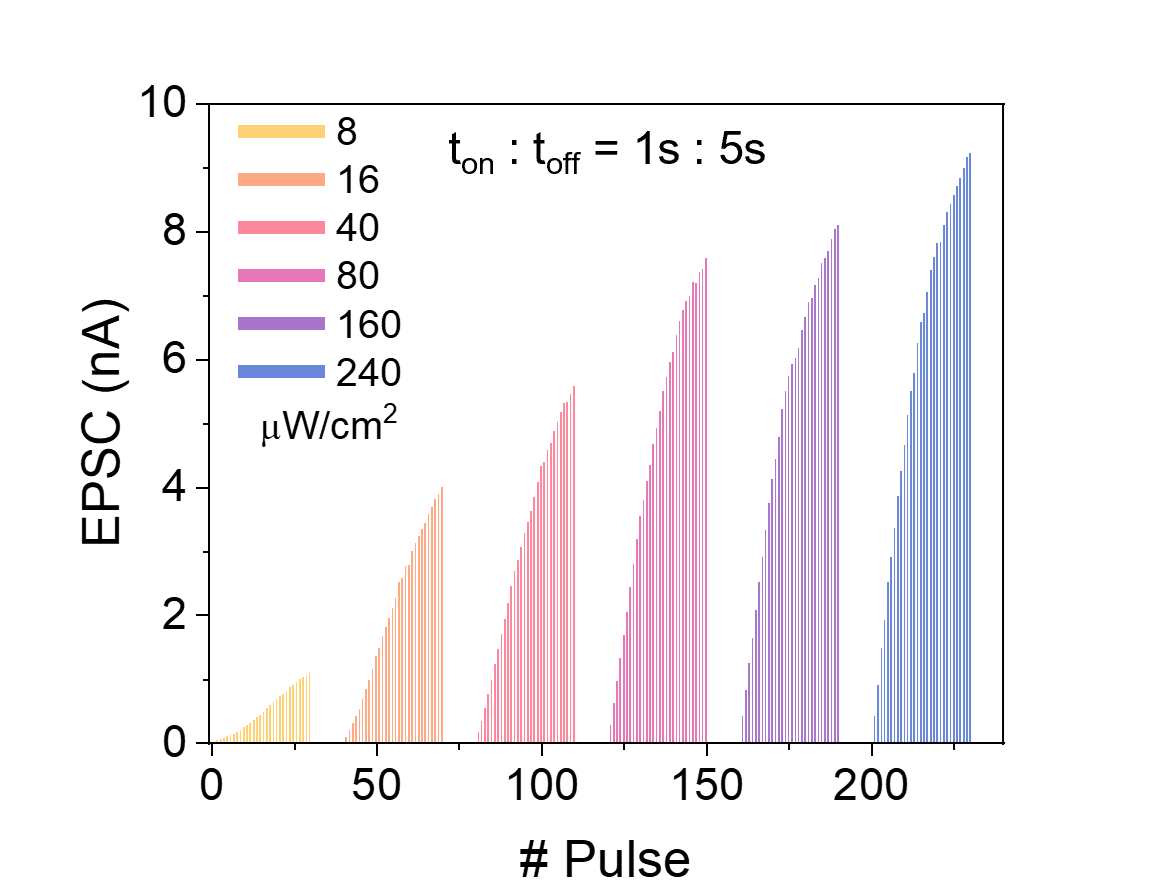


Figure S10. The EPSC induced by a series light pulse with different light power density.


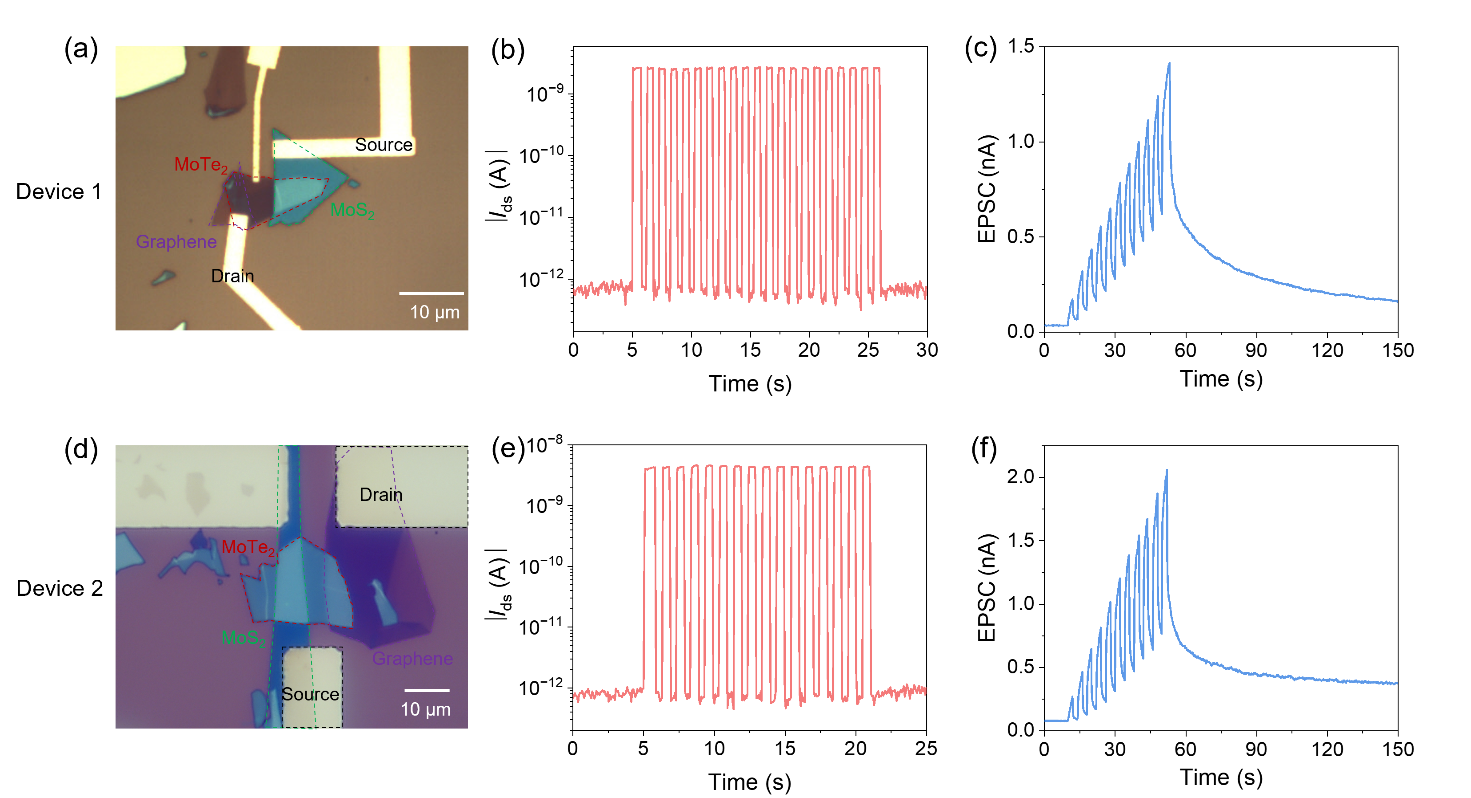


Figure S11. Gate-tunable dual-mode functionality of the MoTe_2_/MoS_2_ vdWH with different MoS_2_ and MoTe_2_ thicknesses. (a, d)Optical microscopy images of the MoTe_2_/MoS_2_ vdWH devices fabricated different thicknesses of MoS_2_ and MoTe_2_ layers. (b, e) Time-dependent photoresponse behavior of the device under 532 nm light with *V*_ds_ = 0 V. (c, f) The EPSC response induced by a series light pulse.


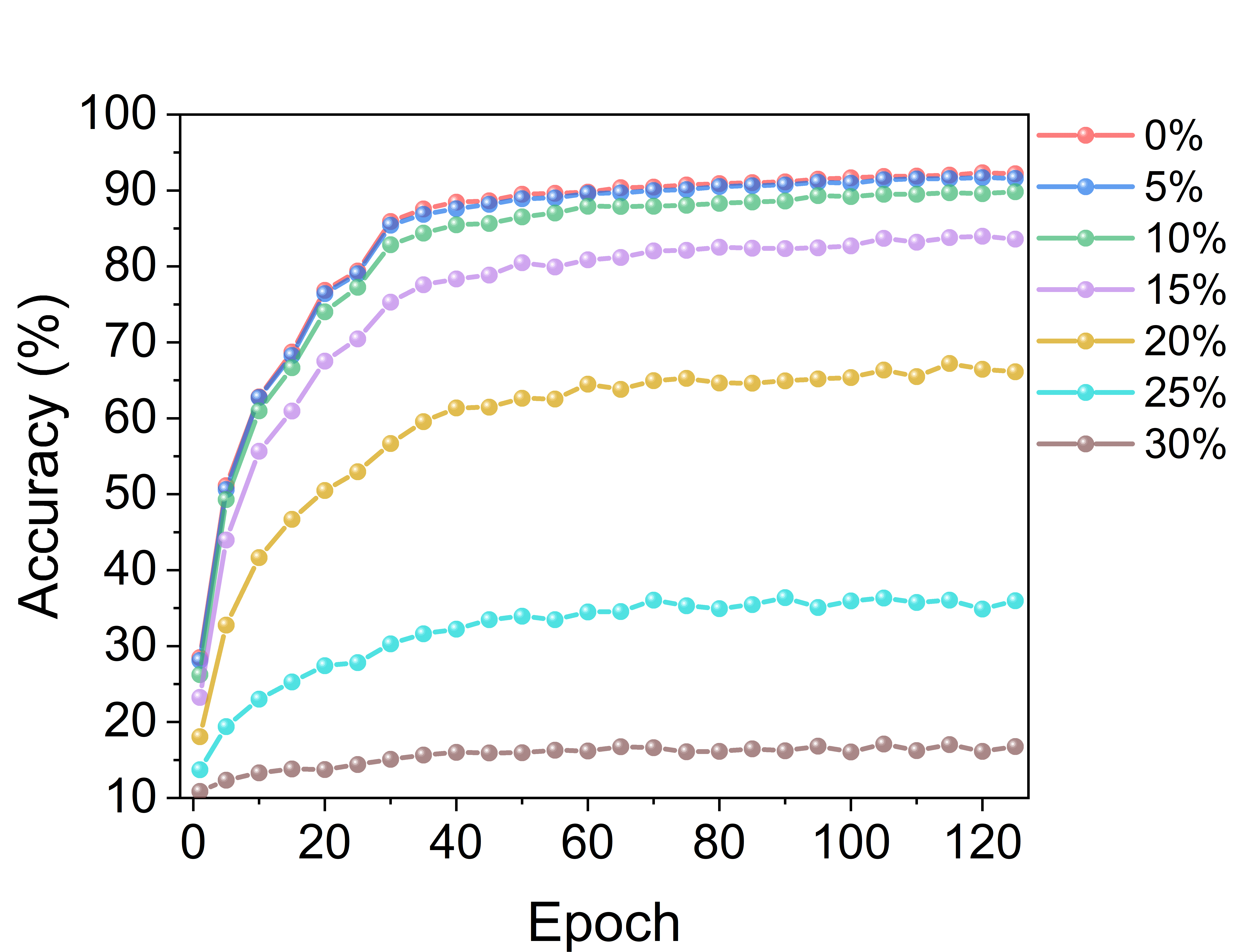


Figure S12. MNIST image classification accuracy of the device under different read noise.

Table S1. Comparison of the self-powered photodetector performance based on MoTe_2_/MoS_2_ vdWH.

| Materials | Thickness  (nm) | V_oc_  (V) | λ  (nm) | R  (mA/W) | τ_r_, τ_f_  (µs) | Ref. |
| --- | --- | --- | --- | --- | --- | --- |
| Gr-MoTe_2_/MoS_2_ | 4.3/4.2 | 0.17 | 365-1550 | 300(532nm) | 188, 45 | This work |
| MoTe_2_/MoS_2_  Dual-floating | 11/11 | 0.4 | 405-1600 | 1570(532nm) | 30, - | [1] |
| MoTe_2_/MoS_2_ | 6.7/74.4 | 0.6 | 532, 1064 | 111(532nm) | - | [2] |
| MoTe_2_/MoS_2_ | 6/7 | 0.45 | 405-1550 | 620(532nm) | 10 | [3] |
| MoTe_2_/MoS_2_ | 3.3/7 | 0.51 | 637 | 46(637nm) | 60, 25 | [4] |
| MoTe_2_/MoS_2_ | 2.2/3 | 0.3 | 470-800 | 322(470nm) | 1000,1000 | [5] |
| MoTe_2_/MoS_2_ | 1.5/3.8 | 0.17 | 473 | 64 | 385 ms, - | [6] |

References

(1) Xu, J.; Luo, X.; Lin, X.; Zhang, X.; Liu, F.; Yan, Y.; Hu, S.; Zhang, M.; Han, N.; Gan, X.; et al. Approaching the Robust Linearity in Dual‐Floating van der Waals Photodiode. Advanced Functional Materials 2023, 34 (12).

(2) Ji, X.; Bai, Z.; Luo, F.; Zhu, M.; Guo, C.; Zhu, Z.; Qin, S. High-Performance Photodetectors Based on MoTe2–MoS2 van der Waals Heterostructures. ACS Omega 2022, 7 (12), 10049-10055.

(3) Ahn, J.; Kang, J. H.; Kyhm, J.; Choi, H. T.; Kim, M.; Ahn, D. H.; Kim, D. Y.; Ahn, I. H.; Park, J. B.; Park, S.; et al. Self-Powered Visible-Invisible Multiband Detection and Imaging Achieved Using High-Performance 2D MoTe(2)/MoS(2) Semivertical Heterojunction Photodiodes. ACS Appl Mater Interfaces 2020, 12 (9), 10858-10866.

(4) Chen, Y.; Wang, X.; Wu, G.; Wang, Z.; Fang, H.; Lin, T.; Sun, S.; Shen, H.; Hu, W.; Wang, J.; et al. High‐Performance Photovoltaic Detector Based on MoTe2/MoS2 Van der Waals Heterostructure. Small 2018, 14 (9).

(5) Pezeshki, A.; Shokouh, S. H.; Nazari, T.; Oh, K.; Im, S. Electric and Photovoltaic Behavior of a Few-Layer alpha-MoTe2 /MoS2 Dichalcogenide Heterojunction. Adv Mater 2016, 28 (16), 3216-3222.

(6) Wang, F.; Yin, L.; Wang, Z. X.; Xu, K.; Wang, F. M.; Shifa, T. A.; Huang, Y.; Jiang, C.; He, J. Configuration‐Dependent Electrically Tunable Van der Waals Heterostructures Based on MoTe2/MoS2. Advanced Functional Materials 2016, 26 (30), 5499-5506.
